# Domain-centric database to uncover structure of minimally characterized viral genomes

**DOI:** 10.1038/s41597-020-0536-1

**Published:** 2020-06-25

**Authors:** John C. Bramley, Alex L. Yenkin, Mark A. Zaydman, Aaron DiAntonio, Jeffrey D. Milbrandt, William J. Buchser

**Affiliations:** 1 Department of Genetics, Washington University School of Medicine, St Louis, MO 63110 USA; 2 Department of Pathology and Immunology, Washington University School of Medicine, St Louis, MO 63110 USA; 3 Department of Developmental Biology, Washington University School of Medicine in St. Louis, St. Louis, 63110 MO USA

**Keywords:** Comparative genomics, Genetic databases

## Abstract

Protein domain-based approaches to analyzing sequence data are valuable tools for examining and exploring genomic architecture across genomes of different organisms. Here, we present a complete dataset of domains from the publicly available sequence data of 9,051 reference viral genomes. The data provided contain information such as sequence position and neighboring domains from 30,947 pHMM-identified domains from each reference viral genome. Domains were identified from viral whole-genome sequence using automated profile Hidden Markov Models (pHMM). This study also describes the framework for constructing “domain neighborhoods”, as well as the dataset representing it. These data can be used to examine shared and differing domain architectures across viral genomes, to elucidate potential functional properties of genes, and potentially to classify viruses.

## Abstract (summary)

**Table:** 

Measurement(s)	Protein Domain • RNA viral genome • DNA viral genome • protein domain neighborhoods • protein domain cluster
Technology Type(s)	digital curation • bioinformatics method • Cluster Analysis
Factor Type(s)	Viral Genome
Sample Characteristic - Organism	Viruses

Machine-accessible metadata file describing the reported data: 10.6084/m9.figshare.12319631

## Background and Summary

Advancements in sequencing technology and the construction of large, publicly available genomic databases have widely expanded the potential for comparative genomics and discovery. But in viruses and bacteria, even protein-coding genomic regions are difficult to functionally characterize. Take *E. coli*, the best-studied bacteria, where one third of the proteome consists of proteins of unknown function. Here, we ask if (1) genomes can be decomposed into a series of functional building blocks that (2) do not rely on annotated genes and that (3) can be used to classify new species or genes, and if (4) protein *domains* can serve as these building blocks.

Automatically defined protein *domains* provide just such building blocks and allow the decoding of some of this ambiguity across genomes. This approach will be based off of the identification of viral domains using profile Hidden Markov models (pHMM) with HMMER3 http://hmmer.org/, v3.2.1^1^. Unlike sequence alignment, pHMMs are able to link two extremely divergent sequences that belong to the same type of protein domain. We referenced the profile databases PFAM^2^, vFAM^3^, and pVOG^4^. Although vFAM and pVOG have not been updated as recently as PFAM, they include many viral-associated domains not found in PFAM. The contents of these three profile-HMM databases form the “PFAM database” referred to throughout this manuscript. Here, we describe the construction of a reference-virus-complete, genome-wide, domain-based database. Domains are identified from the genome sequence, and domain-based “neighborhoods” are constructed. We describe this new dataset, comprising 9,051 viruses, and show some examples of novel queries to answer new biological questions that can be applied to any genome or set of genomes.

Domain-based approaches have been previously used in functional studies of mammalian genes, characterization and identification of pathogenic viruses, and phylogenetic analysis in bacteria^5–8^. Dissecting the domains of novel proteins has led both to a better evolutionary understanding of the driving forces of the genes^5^, insights into taxonomic characterization and evolution^9,10^ and to the discovery of new enzymatic function^11^. Domain neighborhoods are also being used as tools for species classification^6^ and as an alternative to the standard taxonomic classification of 16s-rRNA sequence^8,12^. The success of domain-based classification in bacteria also has the potential to improve difficult viral classification, since there are no genes conserved across every virus.

A slew of recent papers has leveraged groups of protein domains to try to more broadly elucidate function. These include a secretion resource^13^, bacterial pathogenesis^14,15^, and the study of temperature reactive domains^16^. Both GRAViTy (Genome Relationships Applied to Virus Taxonomy) and ClassiPhage 2.0 are tools for examining taxonomy using pHMM-based or genomic structural methods^8,17^. Another paper^18^, describes a new algorithm, MMSeqs. 2 for improving the throughput of the domain detection. Additionally, metagenomic data is difficult to analyze, and is sometimes simply converted to an approximation of species abundance. Instead, a domain-based approach allows for the preservation of the functional complexity within the metagenome, but with a simpler dictionary and a more complete analysis^19^, which we also enable with this work.

## Methods

### Data acquisition and processing

**Fig. 1:**
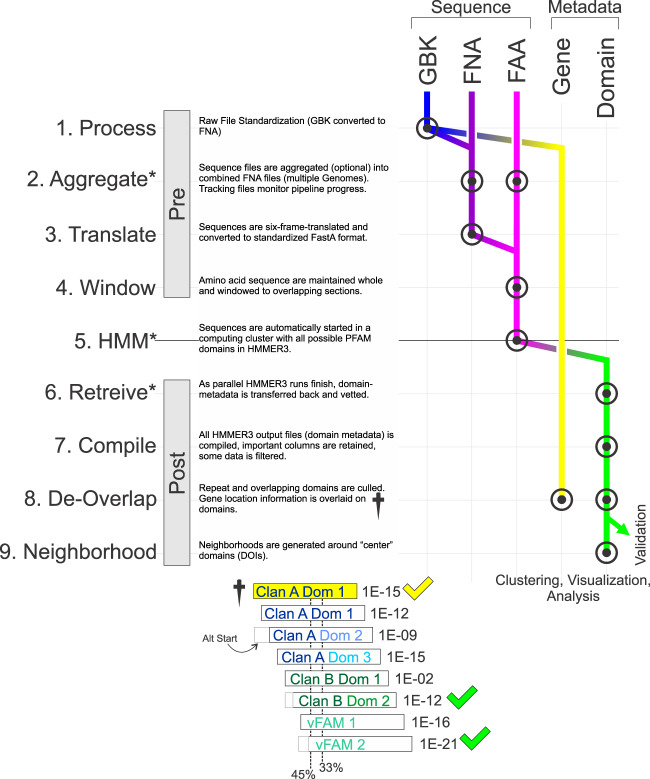
Data Processing Pipeline. On the far left are the steps taken to assemble the datasets in this manuscript. Pre and Post refer to two different custom software that manage the data. Explanations of each step are written in the figure. The diagram on the right shows how different sequence data are processed, and how protein domain metadata is extracted and processed. GBK files are GenBank format, FNA files are nucleotide FastA files, FAA files are amino acid FastA files. Gene metadata includes the name, accession, and genomic coordinates of a gene or open reading frame. Domain metadata includes name, clan, E-value, and genomic coordinates of a protein domain. The de-overlap process (dagger) is shown in the lower panel. This illustrates how the HMMER3 identified domains are curated to filter out duplicate domains that have been over-identified due to the windowing approach. The E-value is listed after an example domain (showing an example clan). The highlighted domain is compared to each overlapping domain to decide on removal of the overlapping domain based on percentage overlap, E-value, and clan. The domains with green checks would be retained and the others would be removed. 45% and 33% overlapping thresholds are displayed.

The data used to build these datasets were retrieved from publicly available sources. 9,051 viral genomes were downloaded from NCBI in the GenBank GBK and FAA format using the NCBI file transfer protocol (ftp.ncbi.nlm.nih.gov/genomes/Viruses/) a full list of accession numbers for the viral genomes used in this work has been included (Accession Number List^20^). The viral genomes include both eukaryotic and prokaryotic viruses spanning a wide range of viral families. While this reference file set will be used as an *example*, the domain-centric workflow is designed to be used with any set of sequence data, including genomic, RNA, protein, and metagenome. The overall pipeline is abstracted in Fig. 1. Each sequence file is processed (Figure 1.1), headers and gene positions are recorded, then the sequences are aggregated (optional) in a standardized FNA (nucleotide FastA) format (Figure 1.2). As each genome is processed and compiled, a tracking file is created and modified to document the progress of each genome through the pipeline (asterisks in Fig. 1). Next, each nucleotide sequence file is six-frame translated with each frame of translation being outputted as a separate file in FAA (amino acid FastA) format (Figure 1.3). The approach of six frame translating genomes and directly searching them enables new un-annotated open reading frames and domains to be found and annotated. All source code is provided (https://gitlab.com/buchserlab/viraldomains).

### Sequence windowing

HMMER3’s *hmmsearch* is sensitive to the length of sequence (target sequence) that is being searched. Our goal was to get a comprehensive look at all the protein domains, even allowing for some overlap (discussed later). Therefore, we used three different approaches to extract domains from each genome. 1) Whole genome search, 2) Gene search, 3) Window search. The whole genome search method simply feeds each entire contig (one of the 6 frames at a time) into *hmmsearch*. In the Gene-based method, we use the existing gene annotations to only feed the identified gene region to *hmmsearch* (open reading frames, ORFs, could also be used in this approach). In the Window method (Figure 1.4), each translated sequence is partitioned into overlapping 200 amino acid ‘windows’. No new sequence information is introduced during this process. Each 200 amino acid segment is then offset by 13 amino acids from the prior segment. As expected, feeding *hmmsearch* smaller sequences (as in the window method) increases its sensitivity to finding established domains compared with providing the algorithm with the entire genomics sequence. A comparison of the genome/window vs. the gene search method is done in the Technical Validation section.

### HMM/HMMER3

The domain profiles used in the pHMM model are from the PFAM, the protein family database provided by the European Bioinformatics Institute (downloaded 2/2018), vFAM, and pVOG databases, totaling 30,947 domains. A complete list of pHMMs is included (pHMM Domains^20^). The vFAM and pVOG databases have been added in order to ensure that any viral domains not included in PFAM have been captured. This database also provides the profile framework for the HMM model^2^. Compiled FAA files are automatically examined to see if they have been previously run, then are copied onto a scratch location in a computing cluster. We then automatically generate new script commands, which run hmmsearch on a computing cluster (Figure 1.5).

The following command is used:

hmmsearch–noali –domT -5 -o /dev/null–domtblout OutName HMMProfiles FAAFile

Where *OutName* is the output file name, *HMMProfiles* is one of 40 pre-compiled profile HMMs (each file contains around 774 individual profiles), and *FAAFile* is the translated genome region that is currently being processed. For a set of genomes, 240 (6 frames x 40 pHMMs) are spawned and run in parallel on the cluster. Profile HMMs were bundled together in order to streamline execution and reduce computational burden. The resulting output files are monitored and if they are complete, they are moved to a different working directory (Figure 1.6). After processing has finished, the tracking logs are updated, but only for the correctly completed files, and the process is repeated, recovering any missing data. Next, the output files from *hmmsearch* (which contain the domain metadata) are compiled together to form a single large table (Figure 1.7). At this step, some filtering is performed. The per-domain independent E-values (iEvalues) are adjusted twice: first, they are scaled to account for the sequence search space size; second, they are scaled again linearly by the ratio of the viral genome size to the window size to adjust for the effects of the windowing. Domains with adjusted E-values > 1 are excluded. While the per-domain bit score and per-domain iEvalue (after accounting for search space size) provide nearly the same information, E-values were used because they are easier to scale, and E-values would most likely be more familiar to a potential researcher using this database. Finally, domains that are 100% overlapping are pruned down to a single copy.

### De-Overlapping

After compilation, the domains are automatically examined to remove spurious results (Figure 1.8). This is mostly from overlapping portions of domains and domains that are part of the same PFAM clan. The steps are, (1) Look at overlap on a per-frame basis (each frame separately), (2) Compare domain start/end and also the calculated start (where the domain would normally start up to 20 AA before). 3a) If neighbor domains are in the same clan, only allow overlap of <33%. 3b) If domains are in different clans, keep both if each are significant; if not, then only allow overlap of <45% (if one domain is 10,000-fold better than the other in E-value). (4) Consider nearest neighbor domains and ‘skip-1’ neighbors (ABC, consider A-B, B-C, AND A-C). In order to address overlapping domains from vFAM/pVOG overtop PFAM, additional logic was constructed. Only in the case that the log10 E-value of the vFAM or pVOG is five times higher than that of the overlapping PFAM domain is the vFAM/pVOG domain preserved.

### Domain neighborhood construction

Next, we want to be able to ask questions of genome neighborhoods at the level of protein domains. Therefore, we want to map these domains onto the genome and reconstruct their ordering (Figure 1.9). There are several concerns to executing this correctly (listed in Usage Notes). The domains are ordered, and the user selects any number of “Domains of Interest”. These domains will act as the center of a genomic neighborhood, and the neighboring domains will list their coordinates in reference to this domain. This step produces the final dataset, and the tables are used to build the domain tracks, clustering, and other figures in the examples.

**Fig. 2:**
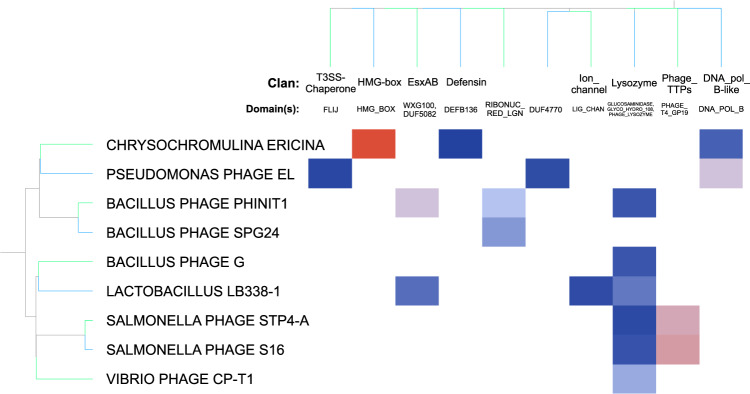
Construction of Clusters. An example (with a restricted set of rows and columns) of how the row clustering is performed. The goal of this clustering is to group related rows together. A row is any grouping of a genomic set of domains (usually a whole virus or a specific virus’s domain neighborhood). For each domain (listed across the top as clans and domains), the inverse square of the domain distance from a domain of interest is used as the value for that column (nearest neighbors would have a value of 1/1^2^ = 1 [blue] and a neighbor 4 domains away would have a value of 1/4^2^ = 0.0625 [red]). If a virus (row) doesn’t contain the clan, the column in that row is assigned a value of 0 (equivalent to a large distance). The result is that rows which have a similar pattern of domains (like the two salmonella phage) are clustered next to each other. 10.6084/m9.figshare.11879253.v1.

The final dataset described above can be explored using a variety of clustering methods. In order to demonstrate this, we used a fuzzy clustering method, by keying the domains by their clan (thus allowing related domains to be grouped) and assigning weights to the domains based on their inverse square distance (ordered domain distance, where Dom1 Dom2 Dom3 the domain distance between Dom1 and Dom3 is 2, rather than amino acid distance) as in Fig. 2. The pre-clustered data is a matrix where domains are columns and viruses are rows. The values are the inverse square distance. So referenced to Domain A, the column for Domain_b_ that is 4 domains away from Domain_a_ in Virus_i_ would get a value of 1/4^2^ = 0.0625. If Domain_b_ is missing in Virus_i_, that cell in the matrix gets a value of 0.

## Data Records

The primary data is available in several tables, available on https://figshare.com/. The first table is comprised of all accession numbers of viral genomes included (Accession Numbers^20^). Next, in (Trimmed Domain Compile File^20^) we provide the table of every domain within each of the reference viral genomes. Examination of domains in close proximity offers insight into conserved structure across genomes and commonly co-occurring domains. The method developed here allows domains found within a genome to be viewed within a “Domain Neighborhood.” The neighborhood comprises domains found in close genomic proximity to one another. This neighborhood is itself often a conserved unit, even when nucleotide sequence conservation is low, similar to genomic synteny, but without relying on primary sequence. The raw tables representing domain neighborhoods are available in (Domain Spacing File^20^). A lookup table containing column descriptors can be found in (Column Lookup Table^20^). A smaller version of the neighborhood file is available as a SQL database containing the necessary tables in (Domain Spacing SQL Database File^20^). Neighborhoods consist of a center *domain of interest*, which takes the zero position, and surrounding domains which have a negative or positive distance values based on whether they are upstream or downstream of the *domain of interest*, respectively. The structure of the data provided allows any domain to be used as the *domain of interest*, enabling the broadest spectrum of potential neighborhoods.

**Fig. 3:**
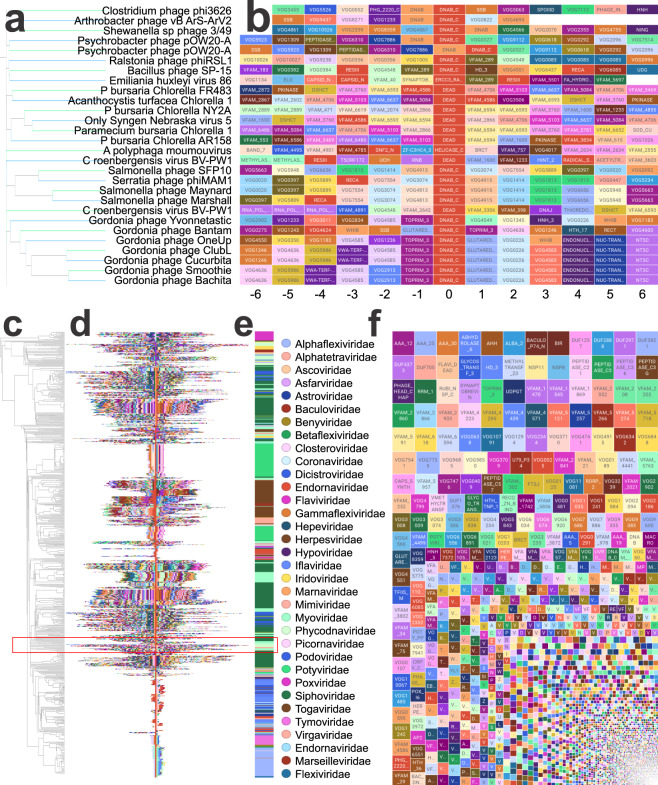
Domain neighborhoods centered around a helicase domain. (**a)** Dendrogram of neighborhoods centered on helicase-associated domains for a set of viruses. **(b)** Domain neighborhoods of genomes that contain helicase-associated domains. The helicase-associated domains are the center (0) positions. Each track (set of domains in a row) corresponds to the virus and dendrogram branch in (**a**) (**c**) Full dendrogram following clustering of helicase domains. (**d**) Domain neighborhood of all helicase-associated-domain-containing genomes. The area enclosed in the red box represents the subset used in panels (a,b). (**e**) Cluster position of viral families throughout the constructed neighborhood. (**f**) Mosaic plot of common domains that co-occur with helicase domains. The size of the block is scaled to demonstrate the sum inverse-square distance of the named domain with the domain of interest (in this case Helicase), with the same metric as mentioned in Fig. 2. This mosaic plot aggregates all of the reference viruses together, thus showing the conserved partners of Helicases (largest domains on the top of the chart). 10.6084/m9.figshare.11879253.v1.

Domain Neighborhoods can be visualized using a domain “track” approach as shown in Fig. 3. Each tile represents a domain upstream or downstream of the center domain. The center domain in Fig. 3 are helicase-associated domains (DNAB_C, DEAD, HELICASE_C, etc). The genomes containing helicase-associated domains in Fig. 3a correspond to the adjacent neighborhoods shown in Fig. 3b. Some conservation can be observed in the domains immediately flanking the center domain (position zero); however, the neighborhoods diverge in more upstream/downstream domains. Figure 3c shows the implementation of the clustering method described above for helicase-associated domains. A broad view of the domain neighborhoods for all genomes that contain helicase-associated domains is shown in Fig. 3d. Using helicase-associated domains as the center domain in clustering resulted in the majority of viruses from the same family being clustered together (Fig. 3e). This data show that by using a fuzzy clustering method, domain neighborhood conservation within viral families can be visualized. In addition to viewing the data as domain neighborhood tracks, mosaic plots can also be used to view domains that commonly occur in the vicinity of the center domain. The size of each tile in the mosaic plot reflects the frequency of the co-proximity with the center domain. In the case of using helicase domains, the most commonly proximal domains are AAA domains (ATPase domains, Fig. 3f).

## Technical Validation

**Fig. 4:**
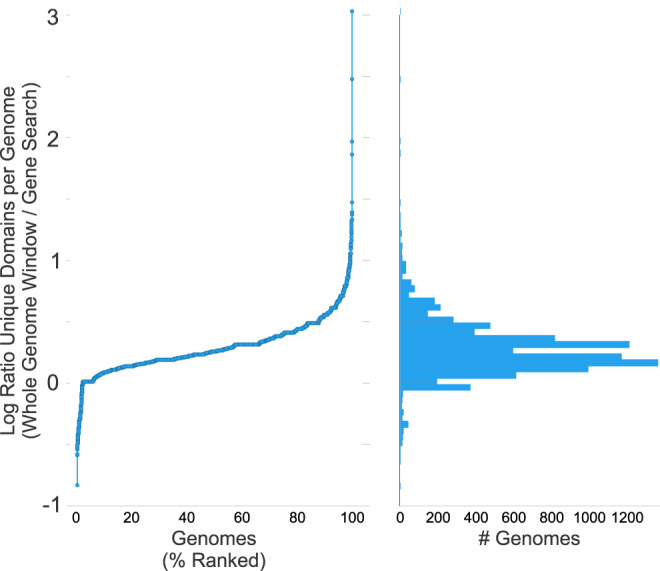
Comparison of Whole Genome vs. Gene Search method for finding domains. Two representations of the distribution of the number of unique domains per viral genome. Higher numbers mean more unique domains were found using the 6-frame translated contigs than the gene/ORF method (as expected). More unique domains are found per genome when using the whole contig versus using genes or identified ORFs. The E-value was the same in each case, and the cut-off was 0.01. 10.6084/m9.figshare.12132762.v1.

**Fig. 5:**
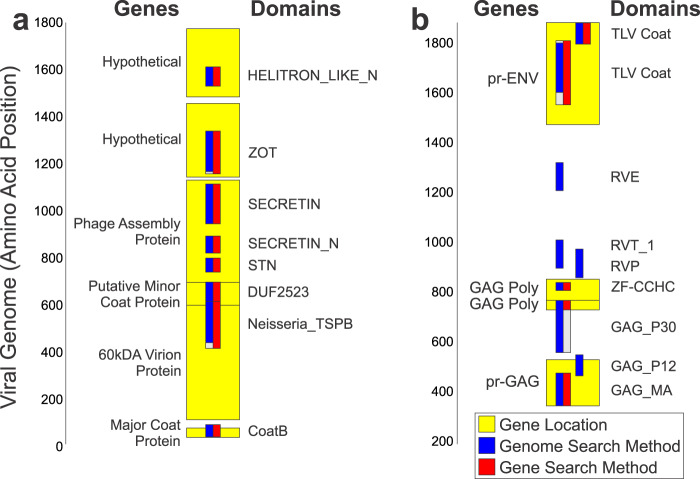
Two example Viral Genomes. Viral genomes showing the annotated coding genes in yellow, and the identified PFAM domains in red and blue. (**a**) NC_001418. “Pseudomonas phage Pf3”, showing representative correspondence between the contig-domain method and the Gene-domain method. (**b**) NC_001500 “Spleen focus-forming virus”, showing the advantage of the contig-based method, specifically that additional high-quality domains are identified outside of annotated coding regions (GAG_P12, RVE, RVT_1, RVP). The start and stop of each domain is demarcated by the bottom and top of the blue and red bars, respectively. The blue bars indicate the domains as identified within the six-frame translated portion of the viral genome’s contig. The red portion shows the domain as identified within the gene. Gray indicates that one of the methods didn’t find the whole extent of the domain compare with the other. In (**b**), there are several domains (GAG_P12, RVE, RVT_1, RVP) that have no corresponding red bar, since no domain was identified with the Gene method. 10.6084/m9.figshare.12132903.v1.

In order to ensure that the domains being identified by our approach are accurate and reproducible our pipeline was validated in two ways. The first validation approach was undertaken to ensure that domain annotation data was properly managed as it progressed through this pipeline. This is critical considering the large scale that HMMER3 is being run and the hundreds of thousands of output files that are produced during this process. In order to ensure that data integrity was maintained, we used the coordinates from the domain metadata of identified domains to extract sequence data from the FNA files. The sequences were reprocessed using the same pipeline to ensure that the same domains were identified. The extracted sequences yielded the same domains that were originally identified. Second, by doing gene/based domain extraction side-by-side with genome/contig based domain extraction, we were able to validate the identity of the domains (Figs. 4 and 5).

Contig-Based Domain-Finding Produces a Rich Set of Functional Domains. After running the pipeline on the set of reference viral genomes, we first sought to determine the completeness of the various methods. Both the translated genome method and the gene method yielded similar numbers of domains. Slightly more domains were found using the whole genome method (Fig. 4) drawn from the “compiled” domain-metadata dataset (Trimmed Domain Compile File^20^). In Fig. 5, two example viral genomes are shown, with gene annotations and the newly annotated domains schematized. Most viral genomes showed good correspondence between identified domains whether looking at the whole genome or looking in genes. Some genomes had gaps of gene annotations, but the genome/contig method was still able to find high-quality domains in these cases (Fig. 5b). Any dataset that relies on gene annotations may have incomplete data, in this case there are ORFs in these positions, but the NCBI database doesn’t have them annotated as genes. Additionally, even lack of ORFs can be misleading (due to pseudogenes and mutations).

Viral genomes are particularly interesting since they are known to perform double coding (overlapping reading frames). An examination of Human Papilloma Virus domains from this dataset showed Domain VFAM_11 and AAA_34 on the positive and negative strand as expected.

**Fig. 6:**
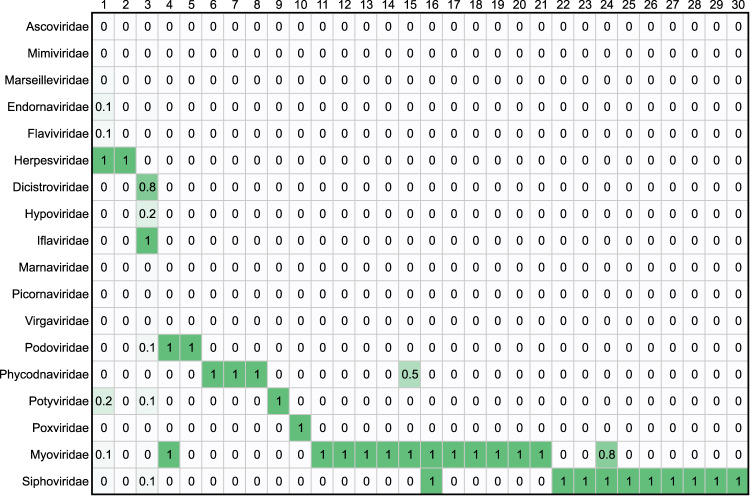
Taxonomic Grouping from Domain Neighborhood Clusters. Contingency matrix of virus family and cluster number. The matrix is scaled to the maximum value on a per-cluster basis. Values closer to 1 are darker. Clusters tend to contain only a single virus family. 10.6084/m9.figshare.11879253.v1.

The goal of this analysis is to redefine any sequence contig as a series of domains and be able to compare those sequences to determine whether they have shared or related domain neighborhoods. This can be used for a variety of purposes, and one of them is to help establish phylogenetic similarity. While this is not the focus of this manuscript, it provides another useful metric to validate the results. By comparing the clustering described above to virus families, we can create a contingency matrix, a scaled version of which is shown in Fig. 6. The adjusted rand index^21^ of these two classifications is 0.53, showing that there is a high statistical correspondence between domain neighborhood-generated clusters and taxonomy. Newer approaches using these types of protein domains are generating exciting connections across biology^22^.

## Usage Notes

### Neighborhood conservation within family-specific domains

**Fig. 7:**
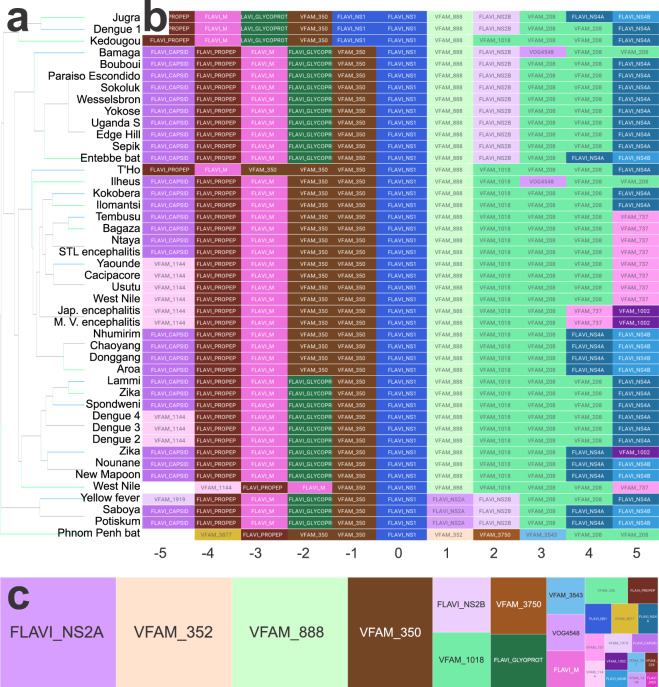
Flavivirus example. (**a**) Dendrogram following unsupervised clustering using the FLAVI_NS1 domain as the center. All genomes containing the NS1 domain were included in the clustering. (**b**) Corresponding domain neighborhood of NS1 containing genomes. NS1 is the center (0) position. Additional FLAVI associated domains are commonly found near NS1 in many genomes. (**c**) FLAVI_NS1 mosaic plot displaying domains commonly occurring with NS1. 10.6084/m9.figshare.11879253.v1.

The data from (Domain Spacing File^20^), in addition to enabling domain comparison using widely present domains, allow for domain examination of family-specific and/or less common domains. This is made possible by using all available PFAM domain profiles as *domains of interest*. Figure 7 uses the Flavivirus NS1 domain as a *domain of interest* to examine a family-specific neighborhood. The flavivirus nonstructural protein 1 (NS1) is a glycoprotein that has a diverse set of functions during flavivirus infection impacting replication, immune evasion, and host vasculature disruption^23^. The wide range of roles attributed to this domain makes it a good candidate for further examination. Figure 7a shows clustered flavi NS1 containing genomes. A high level of domain conservation is seen in the domain neighborhoods surrounding flavi NS1 shown in Fig. 7b. The mosaic plot of the NS1 domain shows that other flavivirus associated with replication and immune evasion co-occur with NS1 (Fig. 7c). Flavi NS2A is most commonly found alongside NS1 (Fig. 7b,c). NS2A has also been shown to be involved in immune evasion, specifically interferon inhibition^24^. This targeted approach to domain analysis allows the user of this dataset to gain insights into family specific domains to direct further research.

### Viral classification using domain neighborhoods

**Fig. 8:**
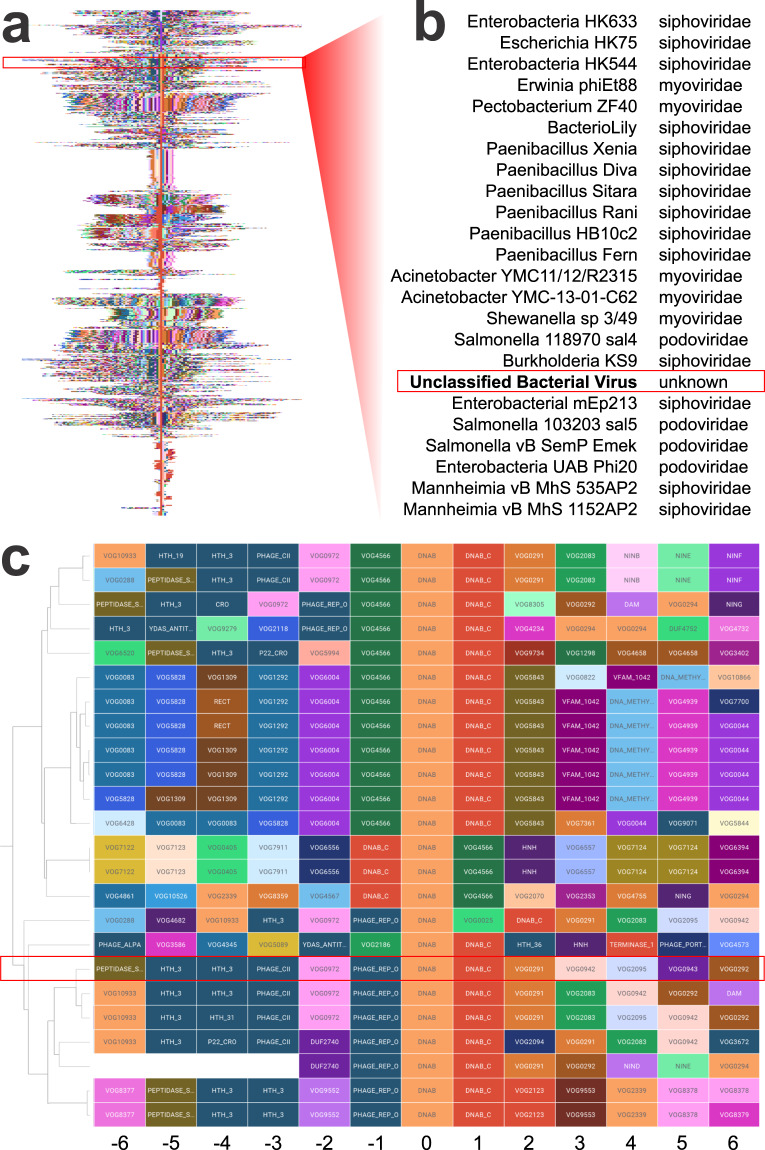
Clustering of Unclassified Phage. (**a**) Genomic neighborhoods of genomes clustered using helicase domains as the center. Zooming in on these neighborhoods reveals genomes characterized as unclassified having a series of close neighbors belonging to the siphoviridae family (**b**). The dendrogram in (**b**) places this unclassified bacterial virus amongst members of the siphoviridae family indicating it could potentially be a member of this family of viruses. (**c**) Further examination of the genomic neighborhood corresponding to the region displayed in (**b**) shows the local domain structure to members of the siphoviridae family. 10.6084/m9.figshare.11879253.v1.

While most of the viral genomes contained in the dataset have been assigned to known viral families, a small subset of the genomes analyzed were recorded as unclassified or unknown with regards to family membership, these will serve as an example of *inferring membership* from these domain neighborhoods. Figure 8a shows the clustered domain neighborhood for all helicase_C-containing genomes. After zooming into a region with an unclassified bacterial virus (Fig. 8b) with the accompanying domain neighborhoods shown in Fig. 8c. Neighborhood-based clustering has placed this virus as a member of the Siphoviridae family. This dataset provides evidence, using a domain-based approach, that this unclassified virus likely belongs to the Siphoviridae family. It is in fact *Enterobacteria YYZ-2008*, a relative of the mEp213 that was clustered next to it (confirmed siphoviridae). Online-only Table 1 provides the nearest neighboring genomes to other unclassified or unknown viruses contained within this dataset. This example shows the potential for using these neighborhoods to infer additional virus’s family membership. These techniques can also be extended to domains of unknown function (DUFs).

We leveraged “BI” software to visualize and organize the domain neighborhoods. We used Tibco Spotfire Analyst for this task, but Microsoft Power BI, Tableau, and other software can also effectively display these datasets. Additionally, Matlab or R (CRAN) can be used.

Below we provide additional concerns on creating domain neighborhoods and domain-based approaches.

1. *Genome Completeness*. We focused our efforts on complete ‘closed’ reference genomes so there would be no question of completeness. If extending these tools beyond viruses to bacteria, some species have additional chromosomes and plasmids which can house the genome neighborhoods. Our software also works on un-assembled genomes (which usually exist as distinct contigs). In these cases, there can be some redundancy and there can also be missing information.
2. *Quality of Domain*. We used *hmmsearch* to identify every possible query domain in the target sequence and reports the iE-value for the profile alignment. We store all these domains but set cutoffs when assembling genome neighborhoods. All domains with E-values less than ~10^−7^ are considered high quality, since a domain in a single genome would be considered high quality with an E-value of less than 0.01, and this threshold is divided by 9,051 to account for the size of the virome database.
3. *Overlapping Domains*. There are two main types of overlapping domains in a genomic region. One type is inherent to the nature of similar domains given that domains in the same clan can often be detected in the same region. This is expected and easy to untangle by taking only the domain with the best E-value for an overlapping region. The second is the result of lower-quality domains being present, or very large domains which can have smaller domains nested inside them. We used the ‘de-overlap’ algorithm (in methods) to address this.
4. *Splicing, Ribosomal Slippage*. Most viruses and bacteria have continuous coding regions, but introns do exist^25^. Although rare, it is also possible that a single domain is split across two frames due to ribosomal slippage^26^. Our program does not currently account for splicing or slippage, so these domains would be missed or would show up with an artificially low E-values (since they could be split up).

## Data Availability

All source code is provided at (https://gitlab.com/buchserlab/viraldomains). The software is designed to be compiled and run with the publicly available DotNetCore, which can be downloaded free with VS Code, or Visual Studio Community Edition.

## Online-only Table

**Online-only Table 1:** Unclassified Virus Families with Similar Viruses and Putative Families.

Accession	Virus Name	Family Left	Family Right	Putative Family
NC_009552	Geobacillus_virus_E2		Siphoviridae	Siphoviridae
NC_009552	Geobacillus_virus_E2		Siphoviridae	Siphoviridae
NC_011356	Enterobacteria_phage_YYZ-2008	Siphoviridae	Siphoviridae	Siphoviridae
NC_011356	Enterobacteria_phage_YYZ-2008	Siphoviridae	Siphoviridae	Siphoviridae
NC_012531	Solenopsis_invicta_virus_3	Tymoviridae		Tymoviridae
NC_024489	Asterionellopsis_glacialis_RNA_virus	Dicistroviridae		Dicistroviridae
NC_027867	Mollivirus_sibericum	Myoviridae		Myoviridae
NC_027867	Mollivirus_sibericum	Iridoviridae	Podoviridae	Iridoviridae
NC_030453	Diaphorina_citri_flavi-like_virus	Flaviviridae		Flaviviridae
NC_030651	Nylanderia_fulva_virus_1	Endornaviridae	Endornaviridae	Endornaviridae
NC_031264	Brucella_phage_BiPBO1	Siphoviridae	Siphoviridae	Siphoviridae
NC_031264	Brucella_phage_BiPBO1	Siphoviridae	Siphoviridae	Siphoviridae
NC_031912	Flavobacterium_phage_Fpv20		Myoviridae	Myoviridae
NC_031914	Flavobacterium_phage_Fpv1	Myoviridae		Myoviridae
NC_031917	Pseudoalteromonas_phage_BS5	Siphoviridae	Siphoviridae	Siphoviridae
NC_032127	Beihai_hepe-like_virus_4		Astroviridae	Astroviridae
NC_032128	Wuhan_spider_virus_2	Iflaviridae		Iflaviridae
NC_032129	Beihai_mollusks_virus_1	Picornavirales		Picornavirales
NC_032206	Beihai_picorna-like_virus_35	Picornavirales		Picornavirales
NC_032221	Hubei_picorna-like_virus_31	Iflaviridae		Iflaviridae
NC_032412	Posavirus_sp	Dicistroviridae		Dicistroviridae
NC_032434	Beihai_mantis_shrimp_virus_5	Dicistroviridae	Picornavirales	Dicistroviridae
NC_032444	Beihai_barnacle_virus_3	Astroviridae		Astroviridae
NC_032445	Beihai_mantis_shrimp_virus_1	Alphatetraviridae		Alphatetraviridae
NC_032446	Beihai_barnacle_virus_4	Dicistroviridae		Dicistroviridae
NC_032456	Beihai_hepe-like_virus_8	Astroviridae		Astroviridae
NC_032464	Beihai_mantis_shrimp_virus_4	Dicistroviridae		Dicistroviridae
NC_032473	Wenzhou_picorna-like_virus_3	Picornavirales		Picornavirales
NC_032530	Beihai_picorna-like_virus_64	Dicistroviridae		Dicistroviridae
NC_032539	Beihai_picorna-like_virus_100	Dicistroviridae		Dicistroviridae
NC_032545	Beihai_picorna-like_virus_32	Iflaviridae		Iflaviridae
NC_032554	Beihai_sipunculid_worm_virus_2	Hepeviridae		Hepeviridae
NC_032556	Beihai_picorna-like_virus_61	Dicistroviridae		Dicistroviridae
NC_032578	Beihai_picorna-like_virus_69		Dicistroviridae	Dicistroviridae
NC_032588	Beihai_picorna-like_virus_93		Hypoviridae	Hypoviridae
NC_032590	Beihai_picorna-like_virus_63	Dicistroviridae		Dicistroviridae
NC_032593	Beihai_razor_shell_virus_1		Dicistroviridae	Dicistroviridae
NC_032611	Beihai_picorna-like_virus_125		Dicistroviridae	Dicistroviridae
NC_032619	Beihai_picorna-like_virus_4		Dicistroviridae	Dicistroviridae
NC_032638	Beihai_picorna-like_virus_91		Iflaviridae	Iflaviridae
NC_032639	Beihai_picorna-like_virus_33		Dicistroviridae	Dicistroviridae
NC_032642	Beihai_picorna-like_virus_82		Dicistroviridae	Dicistroviridae
NC_032755	Changjiang_picorna-like_virus_3		Dicistroviridae	Dicistroviridae
NC_032756	Hubei_picorna-like_virus_40	Iflaviridae		Iflaviridae
NC_032758	Hubei_tick_virus_2		Hypoviridae	Hypoviridae
NC_032759	Hubei_picorna-like_virus_56		Endornaviridae	Endornaviridae
NC_032766	Hubei_orthoptera_virus_1		Dicistroviridae	Dicistroviridae
NC_032769	Hubei_picorna-like_virus_42	Iflaviridae		Iflaviridae
NC_032774	Hubei_diptera_virus_1	Dicistroviridae	Dicistroviridae	Dicistroviridae
NC_032790	Hubei_picorna-like_virus_39	Iflaviridae	Iflaviridae	Iflaviridae
NC_032798	Shahe_endorna-like_virus_1	Myoviridae	Endornaviridae	Myoviridae
NC_032808	Wenzhou_picorna-like_virus_2	Dicistroviridae		Dicistroviridae
NC_032834	Wenling_picorna-like_virus_5	Dicistroviridae	Picornavirales	Dicistroviridae
NC_032874	Changjiang_crawfish_virus_1		Picornaviridae	Picornaviridae
NC_032890	Wenzhou_channeled_applesnail_virus_3		Dicistroviridae	Dicistroviridae
NC_032911	Wenzhou_picorna-like_virus_39	Dicistroviridae		Dicistroviridae
NC_032916	Hubei_picorna-like_virus_17	Picornavirales	Dicistroviridae	Picornavirales
NC_032940	Shahe_picorna-like_virus_1	Picornaviridae		Picornaviridae
NC_032979	Hubei_picorna-like_virus_26	Iflaviridae		Iflaviridae
NC_032989	Wenzhou_shrimp_virus_4		Picornavirales	Picornavirales
NC_032990	Hubei_picorna-like_virus_63	Togaviridae		Togaviridae
NC_033001	Wenzhou_picorna-like_virus_37		Podoviridae	Podoviridae
NC_033003	Hubei_picorna-like_virus_61		Hypoviridae	Hypoviridae
NC_033025	Hubei_picorna-like_virus_38	Dicistroviridae		Dicistroviridae
NC_033030	Hubei_coleoptera_virus_1		Iflaviridae	Iflaviridae
NC_033041	Wenzhou_picorna-like_virus_20		Iflaviridae	Iflaviridae
NC_033061	Hubei_odonate_virus_2		Iflaviridae	Iflaviridae
NC_033094	Hubei_tetragnatha_maxillosa_virus_2	Iflaviridae		Iflaviridae
NC_033099	Hubei_picorna-like_virus_28	Iflaviridae		Iflaviridae
NC_033108	Hubei_picorna-like_virus_32	Dicistroviridae	Iflaviridae	Dicistroviridae
NC_033131	Wenzhou_picorna-like_virus_5		Dicistroviridae	Dicistroviridae
NC_033136	Hubei_picorna-like_virus_43	Iflaviridae		Iflaviridae
NC_033151	Wenzhou_picorna-like_virus_4		Iflaviridae	Iflaviridae
NC_033184	Wenzhou_hepe-like_virus_1	Endornaviridae		Endornaviridae
NC_033194	Sanxia_water_strider_virus_8		Iflaviridae	Iflaviridae
NC_033195	Hubei_picorna-like_virus_35		Iflaviridae	Iflaviridae
NC_033204	Hubei_endorna-like_virus_1	Endornaviridae		Endornaviridae
NC_033205	Wenzhou_picorna-like_virus_26		Dicistroviridae	Dicistroviridae
NC_033206	Hubei_odonate_virus_5	Iflaviridae	Iflaviridae	Iflaviridae
NC_033226	Hubei_picorna-like_virus_51		Iflaviridae	Iflaviridae
NC_033227	Hubei_picorna-like_virus_22		Dicistroviridae	Dicistroviridae
NC_033238	Hubei_picorna-like_virus_41		Endornaviridae	Endornaviridae
NC_033243	Hubei_odonate_virus_4		Iflaviridae	Iflaviridae
NC_033246	Sanxia_picorna-like_virus_3		Dicistroviridae	Dicistroviridae
NC_033262	Sanxia_picorna-like_virus_5		Marnaviridae	Marnaviridae
NC_033419	Wuchan_romanomermis_nematode_virus_1		Hepeviridae	Hepeviridae
NC_033421	Wuhan_arthropod_virus_1		Astroviridae	Astroviridae
NC_033462	Wuhan_fly_virus_4	Iflaviridae		Iflaviridae
NC_033714	Wuhan_spider_virus_5		Virgaviridae	Virgaviridae
NC_033722	Wuhan_insect_virus_33	Dicistroviridae	Dicistroviridae	Dicistroviridae
NC_033825	Bat_badicivirus_1		Iflaviridae	Iflaviridae
NC_034248	Rhizobium_phage_RHEph10	Mimiviridae	Herpesviridae	Mimiviridae
NC_034249	Kaumoebavirus	Mimiviridae	Myoviridae	Mimiviridae
NC_034249	Kaumoebavirus	Phycodnaviridae	Mimiviridae	Phycodnaviridae
NC_034383	Pacmanvirus_A23	Mimiviridae	Phycodnaviridae	Mimiviridae
NC_034622	Alfalfa_virus_S	Alphaflexiviridae	Alphaflexiviridae	Alphaflexiviridae
NC_035465	Morelia_viridis_nidovirus		Coronaviridae	Coronaviridae
